# Subjective craving and event-related brain response to olfactory and visual chocolate cues in binge-eating and healthy individuals

**DOI:** 10.1038/srep41736

**Published:** 2017-02-03

**Authors:** I. Wolz, A. Sauvaget, R. Granero, G. Mestre-Bach, M. Baño, V. Martín-Romera, M. Veciana de las Heras, S. Jiménez-Murcia, A. Jansen, A. Roefs, F. Fernández-Aranda

**Affiliations:** 1 Department of Psychiatry, University Hospital of Bellvitge-IDIBELL, Barcelona, Spain; 2 Ciber Fisiopatologia Obesidad y Nutrición (CIBERObn), Instituto Salud Carlos III, Barcelona, Spain; 3 Addictology and Liaison Psychiatry Department, Nantes University Hospital. Nantes, France; 4 EA 4275 SPHERE “Methods for Patients Centered Outcomes and Health Research”, University of Nantes, France; 5 Department of Psychobiology and Methodology. University Autònoma of Barcelona, Spain; 6 Department of Clinical Sciences, School of Medicine, University of Barcelona, Spain; 7 Clinical Psychological Science, Faculty of Psychology and Neuroscience, Maastricht University, The Netherlands

## Abstract

High-sugar/high-fat foods are related to binge-eating behaviour and especially people with low inhibitory control may encounter elevated difficulties to resist their intake. Incentive sensitization to food-related cues might lead to increased motivated attention towards these stimuli and to cue-induced craving. To investigate the combined influence of olfactory and visual stimuli on craving, inhibitory control and motivated attention, 20 healthy controls and 19 individuals with binge-eating viewed chocolate and neutral pictures, primed by chocolate or neutral odours. Subjective craving and electroencephalogram activity were recorded during the task. N2 and Late Positive Potential (LPP) amplitudes were analysed. Patients reported higher craving than controls. Subjective craving, N2 and LPP amplitudes were higher for chocolate versus neutral pictures. Patients showed a higher relative increase in N2 amplitudes to chocolate versus neutral pictures than controls. Chocolate images induced significant increases in craving, motivated attention and measures of cognitive control. Chocolate odour might potentiate the craving response to visual stimuli, especially in patients with binge-eating.

In our modern society, food is omnipresent; it can be easily purchased and rapidly consumed, without the need of further processing steps. The ubiquity of food in the environment may be highly problematic for some individuals, leading to obesity or even eating disorders. Binge eating disorder (BED) and bulimia nervosa (BN) are both marked by recurrent binges and high experienced craving^1^. Craving is defined as a strong and irresistible desire to consume a specific substance and often leads to loss of control over food intake^2^. External cues such as the sight or smell of food are known to trigger craving and overeating^3^. The purpose of this study was to look at differences in the processing of and reaction to high-palatable food stimuli in patients with binge-eating (BE) when compared to healthy normal-eating adults. This could help to better understand the susceptibility to BE and cognitive processes which may protect from this kind of behaviour.

Chocolate is one of the most heavily craved foods and perceived as highly problematic with regard to the controllability of its intake^4^. However, it is not yet clear how exactly chocolate consumption influences physiological, psychological and biological functioning. There is some evidence suggesting that it is not the pharmacological effect of chocolate alone, nor its high sugar content, which produce these strong cravings in humans, but rather the sensory experience, a combination of different factors such as aroma, caloric content, and texture^5^. So far, however, little attention has been paid to the role of odour in the generation of craving.

With regard to olfactory cue-induced craving, research has shown that the smell of food leads to both intensified craving and increased food intake in restrained as well as in normal eaters^6^. Although, in contrary to taste, food odour is not a primary reinforcer, specific odours might get associated to the taste and reward response of food through conditioning^7^. Furthermore, the combination of odour and taste are known to create flavour and thus together influence the reward value of food in the orbito-frontal cortex^8^,^9^,^10^. There is evidence to suggest that a combination of olfactory and visual stimuli lead to a more powerful craving response than visual stimuli alone^11^. In line with this, a study on reward processing found that a combination of viewing and tasting chocolate led to greater activation in the orbito-frontal cortex than the sum of these two modalities when presented separately, which was denominated as “supralinearity”^12^. These findings indicate that olfaction might be an important player in cue-induced craving and increase the craving response to visual stimuli.

As to the effect of food odours on brain activity, basic studies in humans have reported a reduction of low frequency electroencephalogram (EEG) activity in response to different odours^13^,^14^; for chocolate odour in specific reduced frontal theta activity (4–7 Hz) was found in comparison to other odours or to neutral olfactory stimulation^15^. The authors explained this by a more relaxed state when participants are exposed to chocolate odour; it is however unclear how this reduction in theta frequency may relate to chocolate craving. A recent study found increases in theta power to be related to associative appetitive learning of images to food stimuli^16^; investigating differences in theta power in response to chocolate odour between patients with binge-eating and healthy controls might be helpful to conclude on the importance of odour in stimulus-induced craving.

With regard to visual stimuli, incentive salience of food cues, which corresponds to the craving or “wanting” for these stimuli^17^, has been studied using event-related potentials (ERP) of EEG. The Late Positive Potential (LPP) is an ERP scaling the motivational value of pictures in that more arousing stimuli lead to higher amplitudes^18^. It is enhanced for substance-related stimuli in people with substance use disorders^19^ and can be seen as an indicator of “motivated attention” towards salient stimuli^20^. In a similar way, food stimuli compared to neutral stimuli in general lead to higher LPP amplitudes^21^,^22^, which illustrates their high incentive salience in healthy, normal-weight individuals. A much debated question is therefore, how motivated attention towards food relates to craving and BE. Contradictory results regarding processing of food stimuli in disordered eating were reported in studies using behavioural measures of attentional bias^23^. ERPs have the potential of a more exact timing and it seems that people with overeating or BE compared to normal-eating individuals may have enhanced attention to food in early ERPs, but there is inconsistency regarding later components, such as the LPP (for a review see Wolz *et al*., 2015b).

Low inhibitory control is another important aspect of overeating, loss of control over food intake and addictive behaviours^24^. Therefore, another component which might be helpful for the understanding of food processing in binge-eating is the N2, an early negative-going anterior-frontal ERP. It has been related to the dorsal anterior cingulate cortex and its conflict monitoring function during Go/No-Go tasks^25^,^26^. In passive picture viewing paradigms, the N2 has been associated to stimulus discrimination, the automatic evaluation of affective valence, and arousal^27^,^28^. Another recent study found that N2 amplitudes were associated with the suppression of the emotional response while viewing neutral and negative pictures, which the authors interpreted as an indicator of conflict^29^. However, with regard to the processing of food stimuli, anterior negative deflections in the N2 time-range in two studies showed that higher amplitudes were related to an increased reactivity to food stimuli^30^,^31^. It was furthermore suggested by Asmaro and colleagues that this anterior negative deflection might be related to top-down cognitive control mechanisms over the desire to consume chocolate^31^.

The main aim of this study was to compare BEP to HC with regard to their craving and neurophysiologic response towards visual chocolate stimuli. Thereby it was hypothesized that chocolate pictures would evoke higher craving (self-report) and LPP and N2 amplitudes than neutral pictures (hypothesis 1a) and that there would be a positive relation between self-reported craving and LPP and N2 amplitudes (hypothesis 1b). With regard to group differences, it was hypothesized that BEP compared to HC would have more craving (self-report), more motivated attention (LPP) and less cognitive control (N2) resources (hypothesis 2). Moreover, we expected an increase of state chocolate craving throughout the experiment (hypothesis 3). A second aim was to explore the influence of chocolate odour on the processing of visual chocolate stimuli; we hypothesized that olfactory and visual stimuli would have an additive effect, leading to a potentiated response (hypothesis 4). The third aim was to test if there are differences between BEP and HC in the response to chocolate odour alone. We expected a higher subjective craving reaction towards odour stimuli in BEP than in HC (hypothesis 5); regarding theta frequency, due to the explorative nature of this aim, no directional hypothesis was put forward.

## Materials and Methods

### Participants

The HC group (*n* = 20, age 20–56 years, see Supplementary Table S1 for *means (M*) and *standard deviations (SD*)) was recruited from students of the University of Barcelona and through a snowball system from hospital staff. The BEP group (*n* = 19, age 19–56 years) was recruited from consecutive referrals to the ED unit of Bellvitge University Hospital and diagnosed by means of semi-structured face-to-face interviews to either BN (*n* = 12) or BED (*n* = 7) according to the criteria of the fifth edition of the Diagnostic and Statistical Manual (DSM; American Psychiatric Association 2013). See Supplementary Tables S1 and S2 for a description and comparison of the two groups with regard to socio-demographic and clinical variables.

Exclusion criteria for all participants were: being male, younger than 18 years, current or life-time history of chronic illness (which could influence electrophysiology) or neurological condition (abnormal EEG activity), having used in the last 24 hours psychoactive medication or drugs that may interfere with smell-taste capacity or cortical activity, current substance dependence, lifetime diagnosis of psychotic disorder, functional anosmia (value <16.5 in “Sniffin’ Test”), and pregnancy. Additionally, in the HC group, an exclusion criteria was a lifetime diagnosis of ED, assessed by means of Structured Clinical Interview for DSM-IV Axis I Disorders (SCID-I) (First *et al*. 1997) or being obese (Body Mass Index [BMI] ≥ 30) or underweight (BMI < 18.5).

### Procedure

The study was conducted in compliance with the Declaration of Helsinki (1975) and the Spanish legislation and norms, after revision and approval by the local ethics committee (CEIC Ciutat Sanitària i Universitària de Bellvitge). All participants signed informed consent. The study participation consisted of two parts of approximately one hour each, which were realized either in one or in two separate sessions (50% of HC and 63% of BEP did two sessions). The first part was to check inclusion criteria, participants were weighed and measured (height and head circumference), and were tested regarding their olfactory capacity. In the second part participants filled in a laboratory questionnaire (momentary mood and hunger (1-item Likert scale from 1 to 9), food eaten this day, menstrual cycle, and intake of coffee, alcohol and drugs in the last 24 hours), then the EEG electrodes were placed on the participant’s scalp and she did the experimental task with a duration of 26 minutes. Participants were instructed to have a normal meal two hours before doing the second session and then to refrain from eating until completion of the experiment.

### Study design and experimental task

**Figure 1:**
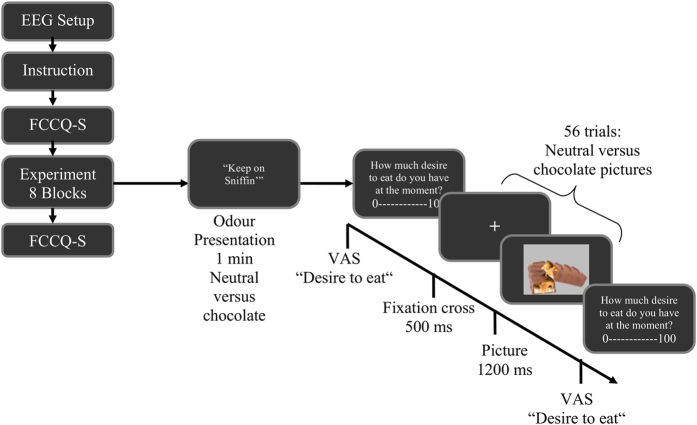
Experimental task and conditions. EEG = Electroencephalogram; FCCQ-S = Food Chocolate Craving Questionnaire; VAS = visual analogue scale.

Hypotheses were tested in a controlled mixed study design. The intra-factors “odour type” and “picture type” were presented at random and counter-balanced order, with 4 combinations of chocolate versus neutral valence in the two sensory modalities (neutral odour - neutral picture, neutral odour - chocolate picture, chocolate odour - neutral picture and chocolate odour - chocolate picture). Each condition was done twice, in two consecutive sections with 8 blocks of 56 trials each, leading to a total of 224 neutral and 224 chocolate picture trials (see Fig. 1).

Visual stimuli were 56 pictures each of chocolate products and neutral office items, used and kindly placed at our disposal by Frankort and colleagues (2014). Pictures were presented in random order on a grey computer screen using the stimulus delivery software Presentation (Neurobehavioral Systems); one block consisted of 56 trials, each starting with a fixation cross (500 ms), followed by the picture (1200 ms). Each visual block was preceded by a 1 min olfactory exposure to either chocolate or neutral odour, presented by a laboratory assistant; the participant had her eyes closed during this exposure. During the olfactory stimulation, participants were exposed to the smell of either a piece of chocolate or a pencil (as in Frankort *et al*.^11^). For a graphical description of the task see Fig. 1.

### Assessment

#### Self-report measures

Subjective chocolate craving was assessed on three different levels: trait craving was assessed during the baseline assessment session and state craving was assessed before and after the experimental manipulation through the *Food Chocolate-Craving Questionnaire* (FCCQ) – State and Trait Version^32^,^33^ (see Supplementary Material, section S1.2., for a more detailed description and psychometrical evaluation of these scales). The momentary craving reaction was assessed through a *visual analogue scale* (VAS) scaling from 0 (very little) to 100 (very strong) asking “At this moment, how much desire to eat do you have?” at 17 time points throughout the experiment, once at baseline and then after each of the odour and visual presentations in the eight blocks.

Additional psychometric questionnaires were used in order to assess difficulties in emotion regulation (Difficulties in Emotion Regulation Scale^34^), food addiction (Yale Food Addiction Scale^35^), eating disorder pathology (Eating Disorder Inventory-II^36^) and general psychopathology (Symptom Checklist-90 revised^37^); olfactory capacity was assessed using the “Sniffin’ Sticks” test^38^ (for a description and reliability measures of the psychometric scales and of the “Sniffin’ Sticks” test see Supplementary Material, section S1).

#### Electrophysiology

EEG was recorded continuously throughout the experimental task using PyCorder (BrainVision). 60 active Ag/AgCI electrodes were inserted into an EEG recording cap (EASYCAP GmbH), distributed after the 10–20 system, the Cz electrode was used as online reference. Four electrodes were placed next to the eyes in order to control for eye movements. Impedances were reduced to be smaller than 20 KOhm using the SuperVisc high-viscosity electrolyte gel for active electrodes. A sampling rate of 500 Hz and an online filter between 0.1 and 100 Hz were used.

The N2 was measured at electrodes AFz (central N2), AF3, F1, F3 (left N2) and AF4, F2, F4 (right N2) as the amplitude and latency of the maximum negative peak in the time window 180–350 ms after visual stimulus onset. The time window for the LPP was set to 300–1000 ms after visual stimulus onset and measured as the maximum positive peak at centro-parietal electrode sites: Pz (central LPP), CP1, CP3, P1, P3, P5 (left LPP) and CP2, CP4, P2, P4, P6 (right LPP). See Supplementary Material, section S2, for a more detailed description of ERP analysis steps and for a description of the analysis of theta power.

### Statistical Analysis

Statistical analyses were conducted with SPSS20 for Windows. For a description of the sample, socio-demographic and clinical variables were compared between the two groups using analysis of variance (ANOVA).

In order to test hypotheses 1a, 2 and 4, for momentary craving in response to picture stimuli, the mean VAS value of the two blocks for each condition was used in an analysis of covariance (ANCOVA) adjusted for baseline momentary craving, with the repeated factors “odour prime” (chocolate/neutral) and “picture type” (chocolate/neutral) and the between subjects factor “group” (HC/BEP). For each of the ERPs (N2 and LPP) the additional factor “localization” was added, wherefore a 2(“odour prime” chocolate/neutral) × 2(“picture type” chocolate/neutral) × 3(“localization” central, right, left) × 2(“group” BEP/HC) ANOVA was calculated for main effects of picture type (hypothesis 1a) and group (hypothesis 2). For hypothesis 4, interaction effects were analysed. Pairwise comparisons were used to follow up main and interaction effects.

Partial correlations including the covariates age and baseline-mood were calculated for each group to look at the relation between dependent measures, i.e. between subjective craving ratings and electrophysiological brain response (hypothesis 1b). Correlations were considered as moderate for *r* > 0.24 (corresponds to *d* > 0.5) and large for *r* > 0.3 (corresponds to *d* > 0.8)^39^,^40^.

For hypothesis 3, state chocolate craving (FCCQ-S) before and after the experimental task was compared using repeated measures (“time” pre/post) ANOVA with the between subjects factor “group” (BEP/HC).

For hypothesis 5 regarding momentary craving in response to odour stimuli, an ANCOVA adjusted for baseline momentary craving, with the repeated factor “odour type” (chocolate/neutral) and the between subjects factor “group” (HC/BEP) was calculated using the mean of the four VAS ratings assessed after odour presentation. For the QEEG data, theta frequency was compared by use of a 2(“odour type” neutral/chocolate odour) × 2(“group” BEP/HC) ANOVA to test for main and interaction effects.

Comparisons were considered significant with *p* < 0.05 after Bonferroni-Finner correction to avoid Type-I errors. Mauchley’s Test was used to test for sphericity and if the assumption was not met (*p* < 0.05), Greenhouse-Geisser corrected values were used. Effect sizes were calculated as partial Eta squared (*η*_*p*_^2^) for ANOVA or Cohen’s *d* for *mean differences (MD*), while *η*_*p*_^*2*^ > 0.01 or *d* > 0.2 are considered as small, *η*_*p*_^*2*^ > 0.06 or *d* > 0.5 as moderate and *η*_*p*_^*2*^ > 0.14 or *d* > 0.8 as large^40^.

## Results

### Ethical Standards

The authors assert that all procedures contributing to this work comply with the ethical standards of the relevant national and institutional committees on human experimentation and with the Helsinki Declaration of 1975, as revised in 2008.

### Baseline measures

Groups did not differ in their olfactory capacity and all of the participants had values within the normal range (TDI > 30 points, see Supplementary Table S1). As expected, BMI was significantly different between groups; it was however not included as a covariate since it is a characteristic of the patient group and therefore is taken into account when comparing the two groups. BEP had higher values than HC in trait craving, difficulties in emotion regulation, food addiction, eating disorder symptomatology and general psychopathology (see Supplementary Table S2). BEP reported lower mood (*MD* = −1.46, *p* < 0.01) and more hunger (*MD* = 1.83, *p* < 0.01) than HC at baseline before doing the experimental task. However, the time since they had had their last meal did not differ between the two groups (*p* = 0.13) and correlations between baseline-hunger and dependent variables (VAS, FCCQ-S total and ERPs) were low (*r*s ≤ 0.3). Age and baseline-mood were both found to have an influence on the correlations between dependent variables, but since the estimated means were very similar (variation of <10%) when adjusting the models by age and baseline-mood into ANCOVAS, in order to simplify the models and to maintain statistical power, it was decided to calculate ANOVAS without adjustments referring to the principle of parsimonia^41^.

### Subjective craving

An ANOVA to compare effects of time and group on state craving showed a significant increase in the FCCQ-S through the experiment, as seen in a significant main effect of time. A significant main effect of group showed that BEP patients reported higher craving than HC at both time points. Apart from the desire and positive reinforcement subscales which did not differ significantly between groups these results were mirrored by all subscales. There were no significant interactions (see Table 1). This supported hypothesis 3 that there would be an increase in state chocolate craving throughout the experiment.

For momentary craving towards visual stimuli, results showed a significant main effect for “picture type” (*F*_*1*,*36*_ = 8.27, *p* < 0.01, *η*_*p*_^*2*^ = 0.19). Chocolate pictures induced higher momentary craving than neutral pictures, which supported hypothesis 1a. A significant main effect of “odour prime” (*F*_*1*,*36*_ = 8.34, *p* < 0.01, *η*_*p*_^*2*^ = 0.19) showed that a preceding chocolate odour led to higher momentary craving than a preceding neutral odour. Pairwise comparisons showed that chocolate compared to neutral odour did not have a significant effect on the rating towards neutral pictures (*MD* = 2.26; *p* = 0.21) but it did affect the rating towards chocolate pictures (*MD* = 5.26; *p* < 0.001), which gave support for hypothesis 4. There was no main effect for “group”, or significant interaction (all *F* < 1.0 and *p* > 0.32), wherefore hypothesis 2 was not supported with regard to higher subjective craving in BEP as compared to HC. See Supplementary Table S3 for *M* and *SD* of momentary craving ratings towards picture stimuli.

Similar to the results for visual stimuli, for the reaction towards odour stimuli a significant main effect of “odour type” (*F*_*1*,*36*_ = 8.34, *p* < 0.01, *η*_*p*_^*2*^ = 0.19) showed more craving in response to chocolate odour than neutral odour, but no main effect of “group” nor an interaction between “group” and “odour type” (*F* = 2.18 and *p* = 0.15) was found, wherefore hypothesis 5 suggesting higher subjective craving in BEP than HC in response to chocolate odour was not supported.

### Electrophysiological data

**Figure 2:**
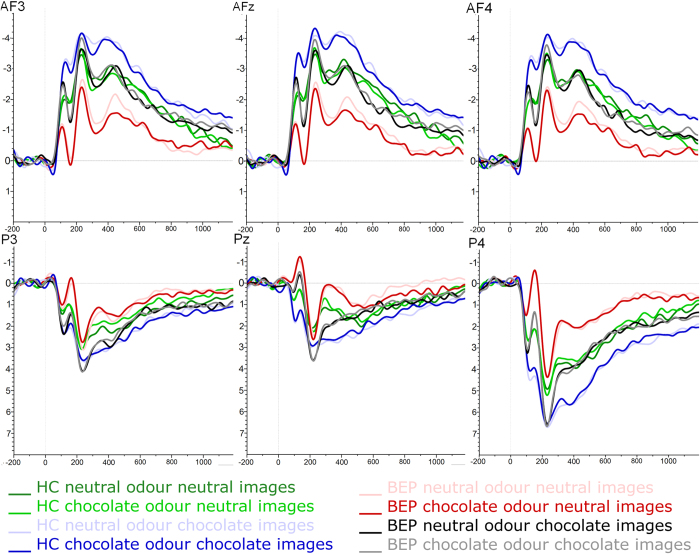
Event-related potentials in response to chocolate and neutral pictures. The graphs show grand averages of stimulus-locked electrophysiological activity from 200 ms before to 1200 ms after stimulus onset. First row: N2 amplitudes (μV) at left (AF3), central (AFz) and right (AF4) anterior-frontal electrode sites. Second row: LPP amplitudes (μV) at left (P3), central (Pz) and right (P4) posterior electrode sites. HC = Healthy control; BEP = Binge-eating patients; LPP = Late Positive Potential.

Two patient data sets had to be excluded from the electrophysiological analyses because of poor data quality. The mean number of segments per condition for the whole sample was between 100 and 102 for all conditions. N2 and LPP mean amplitudes and latencies according to conditions and groups are shown in Table 2 and Fig. 2.

#### N2 peak amplitudes and latencies

For N2 peak amplitudes, there was a significant main effect for “picture type” (hypothesis 1a) and “localization” and a significant interaction effect between “odour prime”, “picture type” and “group” (hypothesis 4); an interaction between “picture type” and “group” was marginally significant (See Supplementary Table S4 for *F*- and *p*-values). No further main or interaction effects were found (all *F* < 2.86 and *p* > 0.10).

The main effect of “picture type” was due to higher negative activity in response to chocolate pictures than to neutral pictures in the whole sample, which was expected by hypothesis 1a. Highest N2 amplitudes were found at the central localization compared to left and right lateralized electrodes; left and right hemispheres did not differ.

**Figure 3:**
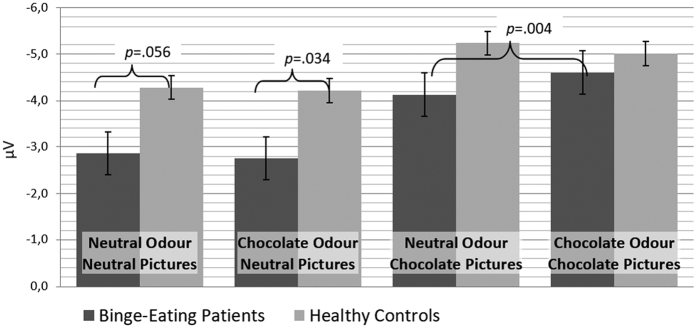
N2 amplitudes (in μV) in response to the presentation of neutral and chocolate pictures for individuals with binge-eating and healthy controls. The graph shows the significant interaction between odour type, picture type and group, which was explained by enhanced amplitudes in response to chocolate pictures preceded by chocolate odour as compared to neutral odour for binge-eating patients, which was not found for healthy controls. Furthermore, there were higher amplitudes during neutral picture processing in healthy controls as compared to patients with binge-eating, but N2 amplitudes in response to chocolate pictures did not differ between groups. Error bars represent standard errors.

The interaction effect between “odour prime”, “picture type” and “group” was explained by BEP having significantly higher amplitudes for chocolate pictures primed by chocolate compared to neutral odour, which was not found for neutral pictures. No such effect was found in HC. Another single effect pointed out by this interaction was seen in higher N2 amplitudes in HC versus BEP for neutral but not for chocolate pictures (see Fig. 3). Therefore, hypothesis 4 of an additive effect of olfactory and visual chocolate stimuli was supported only for the BEP group, but not for HC.

**Figure 4:**
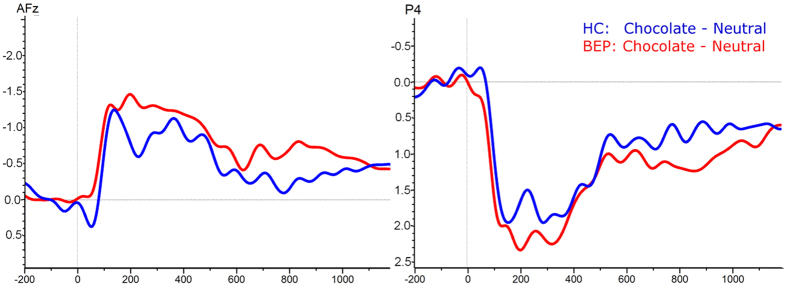
Difference waves for N2 (left panel) and LPP (right panel) amplitudes. Electrophysiological activity during processing of chocolate pictures after subtracting activity during processing of neutral pictures. HC = Healthy control; BEP = Binge-eating patients; LPP = Late Positive Potential.

The marginally significant interaction between “picture type” and “group” was explained through lower amplitudes towards neutral stimuli in BEP than in HC. This led to the conclusion, that BEP have a higher increase in activation when comparing chocolate to neutral stimuli than HC. Therefore, a post-hoc analysis was conducted to calculate a difference score by subtracting for each individual the N2 peak amplitude related to neutral stimuli from the N2 peak amplitude related to chocolate stimuli. An ANOVA of this difference score including “group” as between subjects factor showed a significant effect of “group” (*F*_*1*,*35*_ = 4.95, *p* < 0.05, *η*_*p*_^*2*^ = 0.12), characterized by a higher difference score, i.e. higher relative N2 amplitudes in BEP (*M* = −1.56, *SD* = 0.88) than in HC (*M* = −0.87, *SD* = 0.98). See Fig. 4 for difference waves.

For N2 latency the only significant effect was a main effect of “localization” (see Supplementary Table S4, all other *F* < 2.5 and *p* > 0.09), explained through shorter latencies for the central N2 compared to the left and right lateralized N2 (see Supplementary Table S4).

#### LPP peak amplitudes and latencies

For LPP peak values, there was a significant main effect of “picture type” (hypothesis 1a) and “localization” and a significant interaction between “picture type” and “localization” (see Supplementary Table S4 for *F*- and *p*-values). There were no further main or interaction effects (all *F* < 2.1 and *p* > 0.16). As expected by hypothesis 1a, chocolate pictures led to significantly higher amplitudes than neutral pictures; however, contrary to hypothesis 2, there were no differences between HC and BEP in LPP amplitudes. Highest LPP amplitudes were found at right parietal electrode sites compared to left and central sites, activation at central sites was higher than at left parietal sites (this difference was not significant for neutral pictures) (see Supplementary Table S4 for *MD* and *p*-values).

Regarding LPP latencies, results showed significant main effects for “picture type” and “localization” (see Supplementary Table S4). No further main or interaction effects were found for LPP latencies (all *F* < 2.1 and *p* > 0.16). The LPP peaks for chocolate pictures were earlier in latency than those for neutral pictures. An earlier latency was found for the right LPP compared to central and left LPPs. Latencies did not differ between left and central localization (see Supplementary Table S4).

#### Differences in theta frequency

Contrary to expectations, there were no significant effects for “group” or “odour type” in theta frequency (all *F* < 2.1 and *p* > 0.12).

### Correlation analyses

With regard to hypothesis 1b of a positive relation between subjective and electrophysiological dependent variables, results differed between groups. For HC, momentary craving was positively correlated with N2 and LPP amplitudes, high correlations were found for chocolate pictures primed by chocolate odour. Higher state chocolate craving at baseline went along with higher N2 (*r*s = 0.41 to 0.76) and higher LPP amplitudes (*r*s = 0.12 to 0.48). Surprisingly, for BEP this pattern was different: higher momentary craving (VAS) and higher state craving (FCCQ-S) predicted smaller N2 (*r*s −0.02 to −0.41) and LPP amplitudes (*r*s = 0.06 to −0.41) (see Supplementary Table S5 for *r*-values).

### Post-hoc tests for P1 peaks

Since from visual inspection it appeared that there are big differences between the two groups in P1 amplitudes, a post-hoc analysis with regard to P1 peaks was conducted. Peak amplitudes for the P1 were extracted for each individual at occipital electrodes (Oz and POz) as the maximum positive peak in the time interval 60–150 ms after stimulus onset. An ANOVA including the factors “odour prime” (2), “picture type” (2) and “group” (2) showed a significant main effect of “picture type” (*F* = 42.87 and *p* < 0.001) and of “group” (*F* = 5.57 and *p* < 0.05). There were higher P1 amplitudes for chocolate (*M* = 5.47, *SD* = 0.58) than for neutral (*M* = 2.90, *SD* = 0.51) pictures and HC (*M* = 5.38, *SD* = 0.68) had higher amplitudes than BEP (*M* = 2.99, *SD* = 0.74). Because of these group differences, the analyses for N2 and LPP amplitudes were repeated, including the P1 amplitudes of each condition as covariates. Although *F*- and *p*-values changed slightly by controlling for this factor, in the whole the evidence did not change. The only comparison which lost statistical significance after controlling for P1 amplitudes was the main effect of “localization” for LPP amplitudes. Apart from this, the results found before were supported after taking into account the group differences in P1 amplitudes.

Altogether, the EEG findings support the hypotheses stated in the introduction only partly. Hypothesis 1a of higher LPP and N2 amplitudes for chocolate than for neutral pictures was supported for both the HC and BEP group. With regard to the relation between subjective craving and electrophysiological measures, there were positive correlation coefficients for HC but for BEP hypothesis 1b was not supported. Furthermore, hypothesis 2 was partially supported, since BEP had higher relative N2 amplitudes (difference score) in response to chocolate than HC. However, study results did not show significant differences between groups with regard to LPP amplitudes, wherefore the hypothesis of more motivated attention in BEP compared to HC in response to chocolate stimuli was not supported. Hypothesis 4 of an additive effect of olfactory and visual stimuli was supported only for BEP in that chocolate pictures preceded by chocolate odour led to higher N2 amplitudes than chocolate pictures preceded by neutral odour. The influence of chocolate odour compared to neutral odour stimulation was not visible in theta frequency changes for either of the groups (hypothesis 5).

## Discussion

The current study aimed to compare the subjective craving and event-related brain response of individuals with BE and healthy individuals towards visual and olfactory chocolate stimuli. Additionally, the study aimed to explore the additive effect of olfactory and visual stimuli on craving induction.

The first set of analyses referred to subjective data, showing that both groups had an increase in craving through the experimental manipulation, BEP reported more craving at baseline and after the experiment than HC. When controlling for baseline craving, chocolate pictures evoked a higher momentary craving response than neutral pictures in participants as a whole, but there were no differences between the two groups. These results were mirrored by ERP amplitudes, with higher amplitudes for chocolate than for neutral pictures in both groups.

As expected, there was a significantly higher central N2 for chocolate than for neutral stimuli. There were no group differences in the absolute N2 amplitudes towards chocolate pictures, but the difference in activation between chocolate minus neutral stimuli was higher for BEP than for HC, i.e. the relative increase in response to chocolate pictures after accounting for activation during neutral pictures was higher for patients with binge-eating than for HC. The N2 has been related to response conflict and cognitive inhibitory control in basic research during Go/No-Go tasks^25^,^26^. In studies looking at food processing in mere picture viewing tasks, a comparable increased frontal negativity was found in restrained eaters in response to available food, which was not found in unrestrained eaters^30^. Another study^31^ found an enhanced N2 (labelled as “Anterior Negativity”) for chocolate versus neutral pictures in non-chocolate cravers, which was significantly reduced after chocolate intake. The authors interpreted this effect as top-down cognitive control in non-cravers; they did however not find this effect in chocolate cravers, which seems somehow counter-intuitive. In our study, although in the absolute amplitudes there were no differences between groups for N2 amplitudes towards chocolate stimuli, BEP had lower amplitudes towards neutral stimuli. In accord with the N2 interpretation of Asmaro and colleagues^31^, this interaction effect might indicate that in the patient group there was a higher relative increase in cognitive control in response to chocolate images than in HC. In addition, looking at correlations between craving and N2 amplitudes, in HC self-reported state craving for chocolate at baseline was a strong predictor of higher N2 amplitudes, ratings of momentary craving were also positively related to N2 amplitudes of HC. This could indicate that HC individuals with higher craving use more top-down control in response to chocolate pictures. However, in the patient group there was a tendency for negative relations between self-reported measures of cravings and N2 amplitudes. A possible explanation for the N2 results as a whole might be that there is a non-linear relation between N2 amplitudes and craving. There might be two subgroups of patients: on the one hand those who, similar to HC, have little increase in N2 amplitudes in response to chocolate but experience stronger craving for chocolate (higher ratings in VAS), and on the other hand those who have a high increase in N2 amplitudes related to chocolate pictures and experience lower craving (seen in VAS ratings). However, these results have to be regarded with care and replication is needed in order to confirm this hypothesis.

Regarding motivated attention, a late, right lateralized, posterior component consistent with the LPP was higher in amplitude for chocolate than for neutral pictures. The hypothesis of BEP having more motivated attention towards chocolate stimuli than HC was however not supported by our data. In contrast to earlier findings^42^, no group effects for differences in LPP peak amplitudes towards chocolate pictures were found. However, this former study used a mix of high-caloric food pictures in contrast to chocolate pictures only in our study. Furthermore, the sample was a mere BED sample, while our sample was a mixed sample of patients with BE symptomatology, including BED and BN. Until now, there is no published data looking at motivated attention towards food in BN by use of EEG, and it is possible that they regulate their attention towards food stimuli in a similar way as it was proposed for obese adults^43^. This is supported by the correlations between self-reported momentary craving and LPP amplitudes, which pointed towards a positive association between craving and motivated attention in HC, but a negative association in BEP.

The second aim referred to a potential “supralinearity” or additive effect of olfactory and visual stimuli on craving induction. Results of subjective data partly support this hypothesis, by showing an increased craving response towards chocolate pictures primed by chocolate odour, while chocolate versus neutral odour did not modulate the self-reported craving towards neutral stimuli. The influence of a preceding odour stimulus was not visible in the amplitudes of the visually evoked ERPs (N2 and LPP) in the whole sample. There was however a higher N2 amplitude for BEP towards chocolate pictures preceded by chocolate odour than chocolate pictures preceded by neutral odour, which was not found in HC. This might indicate a higher susceptibility of BEP to the odour of food stimuli. A recent study has shown that after appetitive conditioning, formerly neutral images lead to increased amplitudes of the N2-P3 component^16^. Therefore, a possible explanation of these N2 results might be an association of chocolate odour to its taste and rewarding effect through classical conditioning in BEP. Appetitive conditioning of taste to odour has been shown to take place in the orbito-frontal cortex and thus chocolate odour might potentiate the craving response to visual cues^7^ through supralinearity^12^.

The third study aim was to look at the effect of olfactory stimulation on electrophysiological activity. Similar to the results for visual stimuli, participants reported more craving after smelling chocolate than neutral odour. Contrary to our hypotheses, no differences between groups were found. Furthermore, theta power density was analysed during neutral and chocolate odour presentation, but results did not show any differences between odour types or groups regarding this measure.

Although this study has many strengths, such as a sample of individuals with BE psychopathology, an experimental design and the comparison of subjective and electrophysiological measures, there are some limitations which have to be considered. First of all, the total sample was too small to have enough power to discover complex interactions with small effect sizes, wherefore some group differences may not have been detected. Second, the patient sample was a mixed sample of BN and BED patients; although both patient groups struggle with BE and there is a high cross-over between these diagnostic categories^44^, there may be other processes underlying each one of these disorders which differ between the two diagnostic categories. Future studies should compare these two groups with larger samples in order to see if there are differences in craving induction depending on diagnosis. Regarding the experimental manipulation, two limitations have to be mentioned: first, the lack of differences in theta activity may be due to the participants having their eyes closed during the presentation of the olfactory stimuli, wherefore the increase in alpha activity may disguise the underlying theta activity. Furthermore, in order to look at each sensory modality separately, olfactory and visual stimuli were not presented simultaneously in this experiment, wherefore the real “supralinearity” effect may be underestimated by the results of this study.

This is an important issue for future research. An olfactometer could be used for a simultaneous and precise, event-related presentation of odour stimuli, which may allow a better understanding of the interaction between olfaction and vision in stimulus induced craving. To better understand the meaning of enhanced ERPs in food processing, future studies should also look at neural generators of these potentials. This could also be helpful to inform about possible targets to reduce craving through neuromodulation, as proposed in recent research^45^,^46^,^47^.

The main conclusions of the current study are that chocolate pictures are related to higher amplitudes in electrophysiological measures of cognitive control (N2) and motivated attention (LPP) than neutral pictures; while BEP might have lower baseline N2 activity, they showed a higher relative increase in response to chocolate cues than HC. When considering self-report, although BEP reported more craving than HC at baseline and after the experiment, when controlling for that variable, there were no differences between the two groups in the craving reaction towards chocolate stimuli (visual and olfactory). Furthermore, an additive effect of olfactory and visual stimuli on cue-induced craving was partially supported. Chocolate odour to some extent increased the incentive value and craving reaction towards visual chocolate images in both healthy and BEP, but BEP seem to be more susceptible to this effect.

## Additional Information

**How to cite this article**: Wolz, I. *et al*. Subjective craving and event-related brain response to olfactory and visual chocolate cues in binge-eating and healthy individuals. *Sci. Rep.* **7**, 41736; doi: 10.1038/srep41736 (2017).

**Publisher's note:** Springer Nature remains neutral with regard to jurisdictional claims in published maps and institutional affiliations.

## Supplementary Material

Supplementary Material

## Figures and Tables

**Table 1:** State craving for chocolate measured by the FCCQ-S directly before and after the experimental manipulation in the two study groups.

	Mean									
	HC; *n* = 20		BEP; *n* = 19		Group Effect		Time Effect		Group*Time Interaction	
	Pre	post	Pre	Post	*F*_*1*,*37*_	*p*	*F*_*1*,*37*_	*p*	*F*_*1*,*37*_	*p*
Desire	4.60	8.95	6.16	10.16	2.27	0.140	**66.05**	**<0.001**	1.99	0.735
Positive reinforcement	6.15	7.35	6.83	9.06	1.58	0.217	**22.04**	**<0.001**	1.12	0.166
Negative reinforcement	4.90	6.30	8.47	9.00	**10.90**	**0.002**	**5.49**	**0.025**	1.13	0.295
Lack of control	4.40	4.90	8.32	9.42	**21.14**	**<0.001**	**5.14**	**0.029**	0.73	0.398
Hunger	6.20	8.40	8.63	10.42	**5.17**	**0.029**	**28.63**	**<0.001**	0.30	0.585
Total score	26.30	35.90	38.58	48.37	**12.70**	**0.001**	**56.71**	**<0.001**	0.01	0.942

BEP = binge-eating patients; BMI = body mass index; HC = healthy controls; MD = mean difference. Significant comparisons are marked in bold.

**Table 2:** N2 amplitudes and latencies at central anterior electrodes and LPP amplitudes and latencies at right posterior electrodes for binge-eating patients (BEP) and healthy controls (HC).

	Odour Prime	Picture Type	Peak amplitude (μV)				Latency (ms)			
			HC (*n* = 20)		BEP (n = 17)		HC (*n* = 20)		BEP (n = 17)	
			*M*	*SD*	*M*	*SD*	*M*	*SD*	*M*	*SD*
N2	Neutral	Neutral	−4.28	2.30	−2.86	2.09	246.09	29.61	246.55	27.51
		Chocolate	−5.23	2.42	−4.13	2.14	251.37	44.38	248.85	32.62
	Chocolate	Neutral	−4.22	2.17	−2.76	2.02	241.21	31.17	241.27	22.08
		Chocolate	−5.01	2.36	−4.60	2.28	241.41	29.47	250.69	35.67
LPP	Neutral	Neutral	3.65	2.30	2.46	1.71	509.27	81.35	507.49	84.23
		Chocolate	4.88	2.85	3.80	1.79	447.90	58.53	494.44	126.36
	Chocolate	Neutral	3.40	1.87	2.42	1.53	479.14	73.56	539.11	116.71
		Chocolate	4.96	2.99	3.80	1.56	463.33	64.16	492.10	119.63

MD = mean difference; SD = Standard deviation.
