# Supplementary material for: Subjective craving and event-related brain response to olfactory and visual chocolate cues in binge-eating and healthy individuals
Source: Sci Rep. 2017 Feb 3;7:41736. doi: 10.1038/srep41736 (PMC5290481; doi:10.1038/srep41736)
Supplement: Supplementary Material [file srep41736-s1.pdf]

Subjective craving and event-related brain response to olfactory and visual chocolate cues in binge-eating and healthy individuals.

Wolz, I.[1,2], Sauvaget, A.[3,4], Granero, R.[2,5], Mestre-Bach, G.[1,2], Baño, M.[1,2], Martín-Romera, V.[5], Veciana de las Heras, M.[6], Jiménez-Murcia, S. [1,2], Jansen, A.[7], Roefs, A.[7] & Fernández-Aranda, F[1,2].

[1]*Department of Psychiatry, University Hospital of Bellvitge-IDIBELL, Barcelona, Spain.*

[2]*Ciber Fisiopatologia Obesidad y Nutrición (CIBERObn), Instituto Salud Carlos III, Barcelona, Spain*

[3]*Addictology and Liaison Psychiatry Department, Nantes University Hospital. Nantes, France*

[4]*EA 4275 SPHERE "Methods for Patients Centered Outcomes and Health Research", University of Nantes, France*

[5]*Department of Psychobiology and Methodology. University Autònoma of Barcelona, Spain.*

[6]*Department of Clinical Sciences, School of Medicine, University of Barcelona, Spain*

[7]*Clinical Psychological Science, Faculty of Psychology and Neuroscience, Maastricht University, The Netherlands.*

Corresponding author: Fernando Fernández-Aranda, Ph.D., FAED. Department of Psychiatry and CIBERObn, University Hospital of Bellvitge-IDIBELL, c/ Feixa Llarga s/n, 08907-Barcelona, Spain. Tel. +34-93-2607227; fax. +34-93-2607193 e-mail: ffernandez@bellvitgehospital.cat

Supplementary Material

S1. Assessment of baseline measures

S1.1 Olfactory capacity: The olfactory capacity of participants was measured by use of "Sniffin' Sticks" [1,2], a test of nasal chemosensory performance through pen-like odour dispensers. The sum of the scores from the three subtests assessing odour threshold, discrimination, and identification results in a total score, the TDI-score with a maximum of 48 points. As defined in Kobal et al., 2000 a TDI-score of 30.5 points or more indicates normosmia, a score between 16.5 and 30 points indicates reduced olfactory function termed hyposmia, and less than 16.5 points indicates functional anosmia.

S1.2. Chocolate craving: The *Food Chocolate-Craving Questionnaire* (FCCQ) – State and Trait Version [4,5] is an adaptation of the Food Cravings Questionnaire, which has been validated in English and Spanish, showing good internal consistency and excellent test-retest reliability [6,7]. The FCCQ-T is a multidimensional measure of chocolate craving measuring on a 1-6 Likert-scale intentions to eat chocolate, positive and negative reinforcement, poor control over chocolate, preoccupation with chocolate, craving as a physiological state, emotion-dependent craving, environment-dependent craving, and guilt-loaded chocolate craving. These nine subscales result in a total score for trait chocolate craving. The state version measures momentary desire to eat chocolate, expected positive and negative reinforcement of eating chocolate, expected loss of control and hunger on a 1-5 Likert-scale.

Cronbach's alpha values for the FCCQ-T in the current sample were excellent for the total score (α =.989) and ranged from good (α =.879, positive reinforcement) to excellent (α =.970, control over chocolate) for the subscales. Internal consistency for the FCCQ-S ranged from acceptable (α =.700, positive reinforcement subscale) to excellent (α =.917, desire subscale) for the baseline assessment and from good (α =.814, positive reinforcement subscale) to excellent (α =.913, control subscale) for the post assessment.

S1.3. Emotion regulation: The *Difficulties in Emotion Regulation Scale* (DERS; Gratz & Roemer, 2004) is a 36-item self-report measure that assesses the individuals' typical levels of emotion dysregulation across six domains: nonacceptance of emotional responses; difficulties pursuing goal-directed behaviours when experiencing negative emotions; difficulties controlling impulsive

behaviours when experiencing negative emotions; lack of emotional awareness; limited access to emotion regulation strategies; and lack of emotional clarity. Higher values indicate greater difficulties in emotion regulation. The DERS has been found to demonstrate good reliability (Cronbach's α =.93; test–retest reliability over a period ranging from 4 to 8 weeks = .88) and adequate construct and predictive validity and is significantly associated with objective (i.e., behavioural, physiological, and neurological) measures of emotion regulation [8,9]. A Spanish version of the DERS is validated in the Spanish general adolescent population (Gómez-Simón, Penelo, & de la Osa, 2014), and in healthy and ED adults [10].

Internal consistency in the current sample for the DERS total score was α = .947 and ranged from good (α = .860, awareness) to excellent (α = .943, non-acceptance) for the subscales.

S1.4. Addictive eating: The *Yale Food Addiction Scale*-Spanish Version (YFAS-S; [11,12]) was used to measure addictive eating patterns using 25 items which are assigned to seven scales, referring to the seven criteria for substance dependence defined by DSM-IV [13]. The diagnosis of food addiction is given when at least three of the seven criteria are fulfilled for a period of the last 12 month and the person feels significantly impaired and/or suffers due to the described behaviour. The YFAS was translated into Spanish and validated in the Spanish adult and ED population, with good validity and reliability scores [12]. Internal consistency for the YFAS in the current sample was excellent (α = .968).

S1.5. Eating disorder pathology: The *Eating Disorders Inventory-2* (EDI-2; Garner et al., 1983) is a 91-item self-report questionnaire that assesses characteristics of ED on the dimensions drive for thinness, bulimia, body dissatisfaction, ineffectiveness, perfectionism, interpersonal distrust, interoceptive awareness, maturity fears, asceticism, impulse regulation, and social insecurity. This scale has been validated in the Spanish population (Garner, 1998), obtaining a mean internal consistency of α =.63. In the current sample, internal consistency values of the total score was excellent (α = .979).

S1.6. General psychopathology: The *Symptom Check-List 90 revised* (SCL-90-R; Derogatis, 1994) is a 90-item self-report questionnaire measuring psychological distress and psychopathology. The items load on nine symptom dimensions: somatization, obsessive-compulsive, interpersonal sensitivity, depression, anxiety, hostility, phobic anxiety, paranoid ideation and psychoticism. The "Global Severity Index" (GSI) is a widely used index of psychopathological distress. The SCL has been validated in the Spanish population obtaining a mean internal consistency of α =.75 (Derogatis, 2002). In this sample, Cronbach's alpha for the total score (GSI) was excellent (α = .988).

S2. Electrophysiological Analysis

All EEG data sets were reviewed by a trained neurologist for any abnormality in the standard EEG recording. The background EEG activity and the response to eye opening were normal. No abnormal EEG activity was detected. After inspection, data was prepared for analysis using the BrainVision Analyzer software (Brain Products GmbH). First, the sampling rate was reduced to 256Hz, whereupon data was re-referenced to an average reference and filtered using a high pass cut-off of 0.1Hz (slope 24dB/oct), a low pass cut-off of 30Hz (slope 24dB/oct) and a notch-filter of 50Hz. After this, each individual data set was searched for artefacts, eye movements were corrected by help of an ocular correction independent component analysis. Two patient data sets had to be excluded from the electrophysiological analysis because of low data quality.

For ERP analyses, data sets were cut into equal epochs starting 200ms before visual stimulus onset until 1200 ms after stimulus onset and baseline corrected (-200 to 0ms). For data sets with at least 40 epochs per condition, epochs were averaged according to the four conditions, and the maximum peak amplitudes and latencies were searched using the semi-automatic mode of BrainVision Analyzer. The N2 was measured at electrodes AFz (central N2), AF3, F1, F3 (left N2) and AF4, F2, F4 (right N2) as the amplitude and latency of the maximum negative peak in the time window 180-350 ms after stimulus onset. The time window for the LPP was set to 300-1000 ms after stimulus onset (as in [14]) and measured as the maximum positive peak at centro-parietal electrode sites: Pz (central LPP), CP1, CP3, P1, P3, P5 (left LPP) and CP2, CP4, P2, P4, P6 (right LPP). Mean values for the respective electrodes at anterior-frontal and centro-parietal electrode sides for each of the left and right clusters were calculated to compare lateralization effects of the N2 and LPP, respectively.

For the quantitative analysis of the electrophysiological data during odour presentation, the four 1-minute epochs of each condition (neutral and chocolate) were cut into segments of 2 seconds. The power density at frontal electrodes (Fz, FCz, F1, F2) in the frequency range of 4-8 Hz (theta) was extracted using Fast Fourier Transformation (FFT) with a Hanning Window of 10% and a resolution of 0.5Hz. The segments were then averaged for each condition separately, whereupon a mean value of the four frontal electrodes was calculated.

Table S1. **Sample characteristics.**

		HC; *n*=20		BEP; *n*=19		*p*
Age (in years)	*Mean, SD*	30.00	9.01	35.00	9.57	.101
BMI (kg/m^2)	*Mean, SD*	21.99	2.81	31.19	10.51	.001
Civil status	*Single*	16	80.0%	13	68.4%	.319
	Married	3	15.0%	6	31.6%	
	Divorced	1	5.0%	0	0.0%	
Olfactory capacity (TDI)	*Mean, SD*	34.99	2.88	33.72	1.70	.105

BEP = binge-eating patients; BMI = body mass index; HC = healthy controls; SD = standard deviation; TDI = Threshold Discrimination and Identification Score of the "Sniffin' Stick" Test.

Table S2. Comparison of clinical variables between groups.

	HC		BEP		
	Mean	SD	Mean	SD	p
Trait Chocolate Craving (FCCQ-T total)	68.15	25.27	132.38	55.50	<.001
Intentions to eat chocolate	4.95	2.33	9.75	4.30	<.001
Positive reinforcement	11.75	5.00	15.88	6.76	.043
Negative reinforcement	5.40	3.00	9.94	5.18	.002
Poor control over chocolate	10.00	5.22	21.94	9.35	<.001
Preoccupation with chocolate	8.63	2.09	19.63	11.06	<.001
Craving as a physiological state	7.25	2.57	13.13	6.17	<.001
Emotion-dependent craving	7.63	3.56	14.81	6.40	<.001
Environment-dependent craving	8.58	4.17	15.38	6.79	.001
Guilt-loaded chocolate cravings	4.35	2.21	11.94	4.33	<.001
Emotion regulation (DERS total)	64.47	20.49	117.59	22.47	<.001
Non-acceptance of emotions	10.79	5.49	19.29	5.58	<.001
Goal-directed behaviour	11.11	3.14	17.94	5.45	<.001
Impulse control	8.89	3.07	18.24	6.36	<.001
Awareness of emotions	12.11	3.97	19.29	4.51	<.001
Emotion regulation strategies	12.68	5.45	27.76	7.11	<.001
Emotional clarity	8.89	3.49	15.06	3.82	<.001
Food Addiction (YFAS criteria fulfilled)	1.45	0.60	5.94	1.26	<.001
Eating pathology (EDI-2 total)	24.11	22.24	115.26	38.77	<.001
General Psychopathology (SCL-90R: GSI)	0.37	0.26	1.89	0.67	<.001

BEP = binge-eating patients; DERS = Difficulties in Emotion Regulation Questionnaire; EDI-2 = Eating Disorders Inventory-2; FCCQ-T = Food Chocolate Craving Questionnaire- T; HC = healthy controls; SCL-90R = Symptom Check List – Revised; SD = standard deviation; YFAS = Yale Food Addiction Scale

Table S3. Momentary Craving in response to neutral and chocolate pictures. Means* (M) and standard deviations (SD) for self-reported momentary craving (visual analogue scale from 0-100) in response to picture stimuli preceded by either neutral or chocolate odour in healthy controls (HC) and binge-eating patients (BEP).

Odour prime	Picture type	HC		BEP	
		M	SD	M	SD
Neutral	Neutral	29.70	22.38	36.39	27.75
	Chocolate	44.50	27.42	57.08	33.30
Chocolate	Neutral	33.60	22.26	37.00	27.90
	Chocolate	49.43	27.67	62.66	31.52

* Mean values for each condition averaged over the two blocks are shown.

Table S4. Statistical parameters of significant main and interaction effects and pairwise comparisons for N2 and LPP peak amplitudes (µV) and latencies.

	Factor	$F_{(df)}$	*p*	η_p^2	Pairwise comparison	*MD*	*p*
N2 peak amplitudes	"Picture type"	$_{(1,35)}$ 63.31	<.001	0.64	Chocolate - neutral	**-1.13**	**<.001**
	"Localization"	$_{(1.5,51.9)}$ 17.73	<.001	0.34	Central -left	**-0.37**	**<.001**
					Central - right	**-0.54**	**<.001**
					Right - left	0.17	.137
	"Group"/"Odour prime"/"Picture type" comparing "Odour prime"*	$_{(1,35)}$ 5.51	<.05	0.17	HC/Neut/Neut-HC/Choc/Neut	-0.05	.824
					BEP/Neut/Neut-BEP/Choc/Neut	-0.16	.490
					HC/Neut/Choc-HC/Choc/Choc	-0.21	.119
					BEP/Neut/Choc-BEP/Choc/Choc	**0.45**	**.004**
	"Group"/"Odour prime"/"Picture type" comparing "Group"*				HC/Neut/Neut-BEP/Neut/Neut	-1.40	.056
					HC/Neut/Choc-BEP/Neut/Choc	-1.22	.096
					HC/Choc/Neut-BEP/Choc/Neut	**-1.51**	**.034**
					HC/Choc/Choc-BEP/Choc/Choc	-0.56	.470
	"Picture type"/"Group" comparing "Group"*	$_{(1,35)}$ 4.05	.052	0.1	Neut/HC-Neut/BEP	**-1.46**	**.039**
					Choc/HC-Choc/BEP	-0.89	.234
N2 peak latency	"Localization"	$_{(2,70)}$ 6.21	<.01	0.15	Central -left	**-4.52**	**.009**
					Central - right	**-6.54**	**.003**
					Right - left	2.02	.332
LPP peak amplitudes	"Picture type"	$_{(1,35)}$ 61.23	<.001	0.64	Chocolate - neutral	**0.95**	**<.001**
	"Localization"	$_{(2,70)}$ 24.19	<.001	0.41	Central -left	**0.38**	**.026**
					Central - right	**-0.99**	**<.001**
					Right - left	**1.37**	**<.001**
	"Picture type"/"Localization" comparing "Localization"*	$_{(2,70)}$ 15.11	<.001	0.3	Neut /central-Neut/left	0.31	.057
					Neut/central-Neut/right	**-0.70**	**<.001**
					Neut/Right-Neut/left	**1.01**	**<.001**
					Choc/central-Choc/left	**0.46**	**.024**
					Choc/central-Choc/right	**-1.27**	**<.001**
					Choc/Right-Choc/left	**1.73**	**<.001**
LPP peak latency	"Picture type"	$_{(1,35)}$ 20.63	<.001	0.37	Chocolate - neutral	**-46.49**	**<.001**
	"Localization"	$_{(2,70)}$ 5.26	<.01	0.13	Central -left	12.84	.436
					Central - right	**48.24**	**.003**
					Right - left	**-35.40**	**.025**

BEP = binge eating patients; Choc = chocolate; HC = healthy controls; MD = mean difference; Neut = neutral.
Statistically significant pairwise comparisons (p <.05) are marked in bold.
*Pairwise comparisons for "picture type" are not listed because the difference between chocolate and neutral pictures was significant on all levels of this interaction (all p <.001).

Table S5. Matrix of correlations for ERP amplitudes with VAS ratings of momentary craving and state craving (FCCQ-S) measured at pre-experiment baseline.

			VAS Rating 2 (Pictures)				FCCQ-S					
		Condition	NN	NC	CN	CC	Negative reinforcement	Desire	Positive reinforcement	Lack of control	Hunger	Total score
HC	LPP	NN	.23	.29	**.30**	.24	***.43***	**.38**	**.39**	**.38**	**.38**	***.48***
		NC	.25	**.32**	.27	.26	**.34**	.29	.26	.12	***.40***	**.35**
		CN	.23	**.30**	.23	.26	**.36**	.27	**.37**	.22	**.36**	**.39**
		CC	.29	***.41***	**.38**	**.37**	***.40***	**.34**	**.30**	.28	**.35**	***.41***
	N2	NN	.12	.12	.21	.18	***.65***	***.63***	***.55***	***.57***	***.55***	***.72***
		NC	.19	.21	.23	**.32**	***.56***	***.56***	***.60***	***.45***	***.63***	***.69***
		CN	.13	.14	.20	.22	***.63***	***.66***	***.64***	***.52***	***.67***	***.76***
		CC	**.31**	**.31**	**.34**	***.40***	***.62***	***.66***	***.63***	***.41***	***.70***	***.74***
BEP	LPP	NN	-.02	**-.39**	-.15	**-.33**	.19	**-.33**	-.05	-.07	.16	-.09
		NC	-.14	***-.41***	-.26	**-.35**	.26	-.25	-.14	-.06	.19	-.03
		CN	-.13	-.28	-.24	-.21	.28	-.24	-.12	.19	.14	.03
		CC	-.22	**-.32**	**-.31**	-.29	.25	**-.30**	-.21	.13	.28	.00
	N2	NN	-.22	-.24	-.29	-.09	.06	-.27	***-.41***	.02	**.33**	-.12
		NC	-.19	***-.41***	-.21	-.25	.09	-.17	**-.32**	-.20	.22	-.15
		CN	.06	-.04	-.05	.13	.12	.15	-.10	.02	.28	.15
		CC	-.21	**-.32**	-.21	-.19	.04	-.01	-.25	-.14	.16	-.07

BEP = binge-eating patients; HC = healthy controls;

NN: neutral odour prime – neutral pictures. NC: neutral odour prime – chocolate pictures. CN: chocolate odour prime - neutral pictures. CC: chocolate odour prime – chocolate pictures.

High correlation coefficients (*r* >.3) are indicated in bold. Very high coefficients are indicated in bold italic (*r* >.4).

References

1. Kobal, G. *et al.* 'Sniffin' sticks': screening of olfactory performance. *Rhinology* **34,** 222–6 (1996).
2. Hummel, T. *et al.* 'Sniffin' Sticks': Olfactory Performance Assessed by the Combined Testing of Odor Identification, Odor Discrimination and Olfactory Threshold. *Chem Senses* **22,** 39–52 (1997).
3. Kobal, G. *et al.* Multicenter investigation of 1,036 subjects using a standardized method for the assessment of olfactory function combining tests of odor identification, odor discrimination, and olfactory thresholds. *Eur. Arch. oto-rhino-laryngology* **257,** 205–11 (2000).
4. Rodríguez, S. *et al.* Adaptation of the food-craving questionnaire trait for the assessment of chocolate cravings: validation across British and Spanish women. *Appetite* **49,** 245–50 (2007).
5. Meule, A. & Hormes, J. M. Chocolate versions of the Food Cravings Questionnaires. Associations with chocolate exposure-induced salivary flow and ad libitum chocolate consumption. *Appetite* **91,** 256–265 (2015).
6. Cepeda-Benito, A., Gleaves, D. H., Williams, T. L. & Erath, S. A. The Development and Validation of the State and Trait Food-Cravings Questionnaires. *Behav. Ther.* **31,** 151–173 (2000).
7. Cepeda-Benito, A. *et al.* The development and validation of Spanish versions of the State and Trait Food Cravings Questionnaires. *Behav. Res. Ther.* **38,** 1125–1138 (2000).
8. Gratz, K. L. & Roemer, L. Multidimensional Assessment of Emotion Regulation and Dysregulation: Development, Factor Structure, and Initial Validation of the Difficulties in Emotion Regulation Scale. *J. Psychopathol. Behav. Assess.* **26,** 41–54 (2004).
9. Gratz, K. L., Rosenthal, M. Z., Tull, M. T., Lejuez, C. W. & Gunderson, J. G. An experimental investigation of emotion dysregulation in borderline personality disorder. *J. Abnorm. Psychol.* **115,** 850–855 (2006).
10. Wolz, I. *et al.* Emotion regulation in disordered eating: Psychometric properties of the Difficulties in Emotion Regulation Scale among Spanish adults and its interrelations with personality and clinical severity. *Front. Psychol.* **6,** 1–13 (2015).
11. Gearhardt, A. N., Corbin, W. R. & Brownell, K. D. Preliminary validation of the Yale Food Addiction Scale. *Appetite* **52,** 430–436 (2009).
12. Granero, R. *et al.* Food Addiction in a Spanish Sample of Eating Disorders: DSM-5 Diagnostic Subtype Differentiation and Validation Data. *Eur. Eat. Disord. Rev.* **22,** 389–396 (2014).
13. American Psychiatric Association. *Diagnostic and statistical manual of mental disorders, 4th Edition*. (American Psychiatric Association, 2000).
14. Schupp, H. *et al.* Brain processes in emotional perception: Motivated attention. *Cogn. Emot.* **18,** 593–611 (2004).
